# Photocurable Bioink for the Inkjet 3D Pharming of Hydrophilic Drugs

**DOI:** 10.3390/bioengineering4010011

**Published:** 2017-01-28

**Authors:** Giovanny F. Acosta-Vélez, Chase S. Linsley, Madison C. Craig, Benjamin M. Wu

**Affiliations:** 1 Department of Chemical and Biomolecular Engineering, University of California, Los Angeles, 420 Westwood Plaza, Room 5531, Boelter Hall, P.O. Box: 951592, Los Angeles, CA 90095-1592, USA; gacosta3@g.ucla.edu; 2 Department of Bioengineering, University of California, Los Angeles, 420 Westwood Plaza, Room 5121, Engineering V, P.O. Box: 951600, Los Angeles, CA 90095, USA; clinsle@g.ucla.edu (C.S.L.); mcraig914@ucla.edu (M.C.C.); 3 Division of Advanced Prosthodontics and the Weintraub Center for Reconstructive Biotechnology, University of California, Los Angeles, CA 90095, USA

**Keywords:** 3D printing, pharmaceutical tablets, hyaluronic acid, inkjet printing, Ropinirole, norbornene

## Abstract

Novel strategies are required to manufacture customized oral solid dosage forms for personalized medicine applications. 3D Pharming, the direct printing of pharmaceutical tablets, is an attractive strategy, since it allows for the rapid production of solid dosage forms containing custom drug dosages. This study reports on the design and characterization of a biocompatible photocurable pharmaceutical polymer for inkjet 3D printing that is suitable for hydrophilic active pharmaceutical ingredients (API). Specifically, hyaluronic acid was functionalized with norbornene moieties that, in the presence of poly(ethylene) glycol dithiol, Eosin Y as a photoinitiator, and a visible light source, undergoes a rapid step-growth polymerization reaction through thiol-ene chemistry. The engineered bioink was loaded with Ropinirole HCL, dispensed through a piezoelectric nozzle onto a blank preform tablet, and polymerized. Drug release analysis of the tablet resulted in 60% release within 15 min of tablet dissolution. The study confirms the potential of inkjet printing for the rapid production of tablets through the deposition of a photocurable bioink designed for hydrophilic APIs.

## 1. Introduction

Pharmaceutical dosages are set in the early clinical trial phase and are defined by the amount of active pharmaceutical ingredient (API) with a therapeutic effect in the most number of patients [1]. This “one size fits all” approach fails to account for physiological differences and genomic diversity among the patient population [2]. Consequently, the top 10 highest grossing medications only have a positive therapeutic effect in 4–25 percent of the patients that take them [3]. Personalized medicine aims to provide patients with API dosages tailored to their genomic and pathophysiological profile for improved clinical outcomes. However, changes in the manufacturing of pharmaceuticals tablets are needed to achieve such control over API dosages. Specifically, technology that: (1) can operate at drug dispensing locations, such as pharmacies and hospitals; (2) is economically viable; (3) dispenses precise drug dosages; and (4) has a short production time is required. 3D printing is one such technology. 3D Pharming—the direct printing of pharmaceutical tablets [4]—offers a viable alternative to fabricate personalized dosage forms over traditional tablet manufacturing techniques, such as direct powder compression where the best case is that a tablet can be split into smaller sections as a way to individualize dosages. Indeed, previous studies have used 3D printing technologies to control a drug’s location within a tablet, the drug release kinetics, and to load multiple APIs within a single tablet [5,6,7,8].

The aim of this study was to engineer a photocurable bioink suitable for the inkjet printing of pharmaceutical tablets loaded with a hydrophilic API. Inkjet printing for 3D Pharming is advantageous for a number of reasons, including its precise dosage control, high spatial resolution, and the ability to monitor droplet dispensing using high-speed cameras for quality assurance [9,10,11]. Additionally, inkjet printing offers fast printing speeds and the ability to print at room temperature for the preservation of temperature labile APIs, which is an advantage over 3D printing techniques previously used for 3D Pharming, such as powder bed printing and fused deposition modelling (FDM), respectively.

Hyaluronic acid (HA), a glycosaminoglycan typically found in connective, neural, and epithelial tissue [12,13], was functionalized with norbornene moieties and used as the main backbone of the photocurable hydrogel precursor solution. This photocurable polymer solution was dispensed into blank preform tablets, made by direct powder compression, and subsequently exposed to visible light to induce rapid polymerization. Finally, the rapid release of the hydrophilic API from the resulting tablet was characterized. The high specificity of the thiol-norbornene reaction preserves the stability of the API loaded in the formulation throughout the gelation process, given the absence of thiols and reactive carbon-carbon double bonds (enes) within the model hydrophilic drug [14,15,16]. For this study, Ropinirole, a non-ergoline dopamine agonist for the treatment of Parkinson’s disease and restless legs syndrome [17], was utilized as model drug due to its high hydrophilicity.

## 2. Materials and Methods

### 2.1. Norbornene Functionalized Hyaluronic Acid (HANB) Synthesis

Hyaluronic acid (HA) was functionalized with norbornene moieties in a two-step synthesis. Briefly, hyaluronic acid (60 kDa·MW) (Genzyme Corporation, Cambridge, MA, USA) (2 g, 5.27 mmol) was dissolved in 400 mL of DI water. Adipic acid dihydrazide (ADH) (18.37 g, 105.45 mmol) was added to the HA solution and the pH was adjusted to 4.75 with hydrochloric acid. 1-ethyl-3-(dimethylaminopropyl) carbodiimide hydrochloride (EDC) (4.05 g, 21.09 mmol) was added to the reaction while maintaining the pH at 4.75. Once the pH stabilized at 4.75, the reaction was allowed to run overnight. The solution was dialyzed for 3 days against DI water (Fisherbrand regenerated cellulose, MWCO 12,000–14,000 Da, Houston, TX, USA). The HA functionalized with hydrazide groups (HA-ADH) was lyophilized and stored at −20 °C until further use. HA-ADH was dissolved in 400 mL of DI water and the pH was adjusted to 7.0. Cis-5-norbornene-*endo*-2,3-dicarboxylic anhydride (Sigma-Aldrich, St. Louis, MO, USA) (4.33 g, 26.36 mmol) was dissolved in 100 mL of dimethylformamide, added dropwise into the HA-ADH solution while maintaining the pH at 7.0 and reacted overnight. The norbornene functionalized HA (HANB) was dialyzed against DI water for 3 days, filtered, lyophilized, and stored at −20 °C. The resulting HANB was characterized by proton nuclear magnetic resonance spectroscopy (^1^H NMR) on a Bruker AV300 broad band FT NMR Spectrometer (Billerica, MA, USA). ^1^H NMR shifts of norbornene groups (D_2_O): δ 6.20 (*endo* vinyl protons, 2H), δ 3.46 (bridgehead protons, 2H), δ 3.61 (norbornene α protons, 2H) (Supplementary Materials Figure S1). All chemicals were purchased from Sigma-Aldrich unless otherwise stated.

### 2.2. Photocurable Formula Preparation

HANB was dissolved in phosphate-buffered saline (PBS) and mixed with variable amounts of Poly(ethylene) glycol dithiol (1500 Da, PEGDT). Eosin-Y was added at a concentration of 0.1 mM and Poly(ethylene) glycol (MW 200, PEG200) constituted 10% of the solution. The hydrophilic drug Ropinirole HCL (obtained as a gift from GlaxoSmithKline, Philadelphia, PA, USA) was loaded into the solution at a concentration of 80 mg/mL. The weight percent of HANB in solution (*W*_HANB_), the ratio of PEGDT thiol groups to norbornenes (*r*_ratio_), and the light exposure time (*T*_L_) were manipulated to achieve the desired hydrogel mechanical properties. All the reagents were purchased from Sigma-Aldrich unless otherwise stated.

### 2.3. Gelation and Mechanical Properties

To prepare gels for mechanical testing, 40 µL of solution was pipetted between two Sigmacote functionalized glass slides and exposed to visible light (Volpi, V-lux 1000) at an intensity of 120 mW/cm^2^, resulting in hydrogels with a cylindrical shape. To assess the effect of light exposure time on hydrogel mechanical properties, gels with *W*_HANB_ = 3% and *r*_ratio_ = 0.6 were exposed to visible light for 5, 10, 30, 60, 120, and 180 s. Hydrogels with *r*_ratio_ = 0.6, *T*_L_ = 2 min, and *W*_HANB_ = 2%, 3%, and 4% were fabricated to analyze the effect of HANB weight percent. Finally, hydrogels with *W*_HANB_ = 3%, *T*_L_ = 2 min, and *r*_ratio_ = 0.2, 0.4, 0.6, and 0.8 were made to analyze the effect of crosslinking ratio. The storage modulus (G’) of the hydrogels was measured in a rheometer (Discovery HR-2, TA Instruments, New Castle, DE, USA) with an 8 mm parallel plate (Peltier plate Steel) geometry at a constant strain of 1%. The final formulation for further characterization and drug dispensing had the following parameters: *W*_HANB_ = 3%, *r*_ratio_ = 0.6, and *T*_L_ = 2 min. In-situ photorheology was performed to analyze the gelation kinetics of the hydrogel.

The inverse of the Onhesorge number (*Z*) was calculated to assess the printability and droplet formation capability of the formula Equation (1) [18], where a is the diameter of the printing orifice, ρ is the density, γ is the surface tension, and η is the viscosity of the formulation.

(1) $$Z=\frac{{\left(a\rho \gamma \right)}^{1/2}}{\eta }$$

Viscosity was measured with a rheometer in a 40 mm 2.016° cone plate geometry with shear rate ranging from 10 to 100 Hz. The density of the formula was obtained by weighing an amount of solution and dividing the mass over its predetermined volume. The surface tension was calculated with a tensiometer (Cole-Parmer, Kimble Chase 14818 Tensiometer, Vernon Hills, IL, USA). Equation (2) [19] was used to determine γ, where ***h*** is the distance between menisci, r is the radius of the capillary, ρ is the density of the formulation, and g is the acceleration due to gravity.

(2) $$\gamma =\frac{1}{2}hr\rho g$$

To measure the swelling ratio of the polymerized formula, hydrogels were left in PBS overnight, weighed, and subsequently lyophilized to obtain their dry weight. The mass swelling ratio $\left(Q_{M}\right)$ was calculated by taking the ratio between the mass of the swollen hydrogel ($M_{S})$ and the dry mass of the lyophilized gel ($M_{D}$) in Equation (3) [20].

(3) $$Q_{M}=\frac{M_{S}}{M_{D}}$$

### 2.4. Thiols Consumption

To assess the photopolymerization reaction kinetics, Ellman’s reagent analysis was performed on hydrogels fabricated with *T*_L_ = 5, 10, 30, 60, 120, and 180 s. The unreacted thiol concentration in the hydrogels over light exposure time was calculated.

### 2.5. Preform Tablet Fabrication

The drug-containing bioinks were directly printed into tablet preforms that were fabricated by direct compression technique using microcrystalline cellulose (Avicel(R) PH-103; FMC Corporation; Philadelphia, PA, USA) as the binder, and croscarmellose sodium (VIVASOL(R); JRS Pharma, Patterson, NY, USA) was added as a superdisintegrant (5% (*w*/*w*)). The preform tablets are shown in Figure S2A, and are comprised of two fitting parts—a 100 µL well and a cap—with a locational interference fit. A customized press design was used to make the preform tablets and was machined by Proto Labs, Inc. (Maple Plain, MN, USA). Briefly, Standard B-Type upper punches were modified to produce the positive features of the cap and the well. The lower punch was designed to sit flush on a hydraulic press stage and had a tip with a 0.04-inch radius of curvature and a 0.02-inch blended landing. A standard 0.945 die with a 3/16-inch-deep TSM standard taper and an increased die bore dimension of 0.003 inch at the face of the die was used in the manufacturing of the tablets (Figure S2B). The preform tablets (150 mg caps; 300 mg wells) were compressed at different compression pressures (1–10 kN), with a dwell time of 30 s. To prevent dissolution of the preform tablet during the printing of the drug-containing bioink, the well was brush-coated with Eudagrit(R) E100 (Evonik, Essen, Germany) (polymethacrylate copolymer) dissolved in acetone at 20% (*w*/*w*) E100. The tablet hardness was measured using Buehler Vicker Hardness indenter 1600-6305 (Buehler, Lake Bluff, IL, USA) with Vickers indentations from 200 to 500 g load.

### 2.6. Drug Dispensing and Release Kinetics

The formula was filtered through a 0.22 µm filter (Fisherbrand) and dispensed into preform tablets (71.25 µL for 5.7 mg of Ropinirole HCL) by using a piezoelectric dispenser with an 80 µm diameter nozzle (MJ-ABP-01-080, MicroFab Technologies, Plano, TX, USA). A dispensing system (MD-E-3000, Microdrop Technologies, Norderstedt, Segeberg, Germany) was used to drive the piezoelectric nozzle, set at 45 V, with a pulse width of 16 µs and a frequency of 2000 Hz. Droplet formation and continuous monitoring of the process was done with an analog camera (JAI CV-S3300), lens (Edmund Optics, Barrington, NJ, USA), and an LED light controlled by the Microdrop driver. After dispensing the formula into preform tablets, the solution was exposed to light for 2 min at 120 mW/cm^2^ to induce polymerization and the tablets were capped. In vitro drug release was measured by placing the tablets inside Uni-cassettes (Tissue-Tek) and submerging them in 200 mL of dissolution medium (2.9 g/L of sodium citrate dihydrate and 3.3 g/L of anhydrous citric acid in water at a pH of 4.0). The dissolution test was conducted at 37 °C and stirred at 60 rpm. 1 mL aliquots were taken after 5, 10, 15, 30, 60, and 120 min of dissolution. The volume removed was replaced with fresh dissolution medium to keep the total volume constant. The aliquots were centrifuged, filtered, and the Ropinirole HCL content was measured by HPLC analysis (Prominence Modular HPLC, Shimadzu, Nakagyō-ku, Kyoto, Japan).

### 2.7. Hydrogel Drying

To improve the stability of the API loaded within the hydrogel for prolonged storage, water must be removed from the gel to avoid possible degradation of the therapeutic compounds. The time required to remove the water content of the hydrogels was calculated by exposing hydrogels to 50 °C for up to 3 h and monitoring the weight of the gels over time.

### 2.8. Statistical Analysis

Statistical analysis was performed with GraphPad Prism software (GraphPad Software, Inc., San Diego, CA, USA). Statistical significance was assessed using single factor ANOVA test with a Tukey post-test and 95% confidence interval. A Dunnett’s post-test analysis was performed on Figure S3 to assess statistical significance.

## 3. Results and Discussion

### 3.1. HANB Synthesis and Gelation

**Figure 1:**
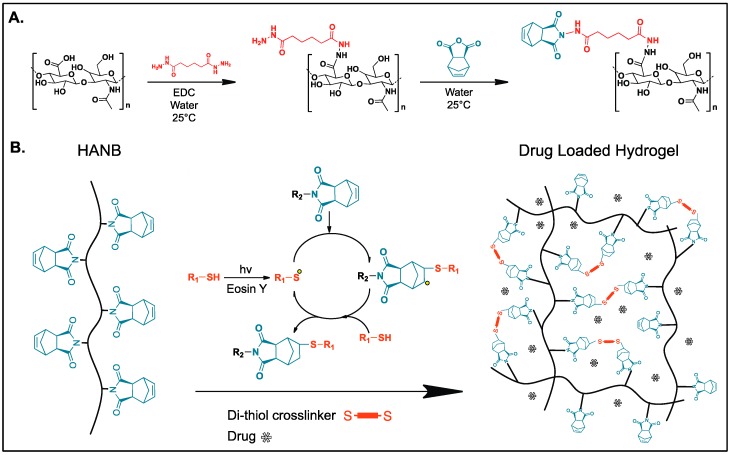
(**A**) Norbornene functionalized hyaluronic acid (HANB) two-step synthesis pathway; (**B**) Bioink photopolymerization for drug loaded hydrogel formation through thiol-ene reaction.

Previously, Gramlich et al. modified HA with norbornene groups by converting it to its tetrabutylammonium salt and further reacting it with 5-norbornene-2-carboxylic acid in the presence of di-tert-butyl dicarbonate, achieving a degree of modification of 20% [21]. In this study, HANB was synthesized in a two-step process (Figure 1A). First, the carboxyl groups in HA were conjugated with ADH by using the carbodiimide EDC as coupling agent. Subsequently, Cis-5-norbornene-*endo*-2,3-dicarboxylic anhydride was reacted to the incorporated hydrazide groups. ^1^H NMR analysis revealed that 50% of the HA monomers were functionalized with norbornene groups. This high degree of modification allows for hydrogel formation with low concentration solutions of HANB, which are required to keep the viscosity of the bioink within the printable region for inkjet printers (<20 mPa·s) [22]. HANB was mixed with PEGDT, Eosin Y (photonitiator), and Ropinirole HCL (model hydrophilic drug). The abundant norbornene groups in the HA backbone react with PEGDT in the presence of visible light (120 mW/cm^2^), forming multiple crosslinks through a light induced step-growth polymerization reaction (Figure 1B). Visible light represents a safer light source than UV light to be used for the operation of printing equipment and to maintain the stability of certain UV light labile APIs [23].

### 3.2. Hydrogel Characterization and Droplet Formation

**Figure 2:**
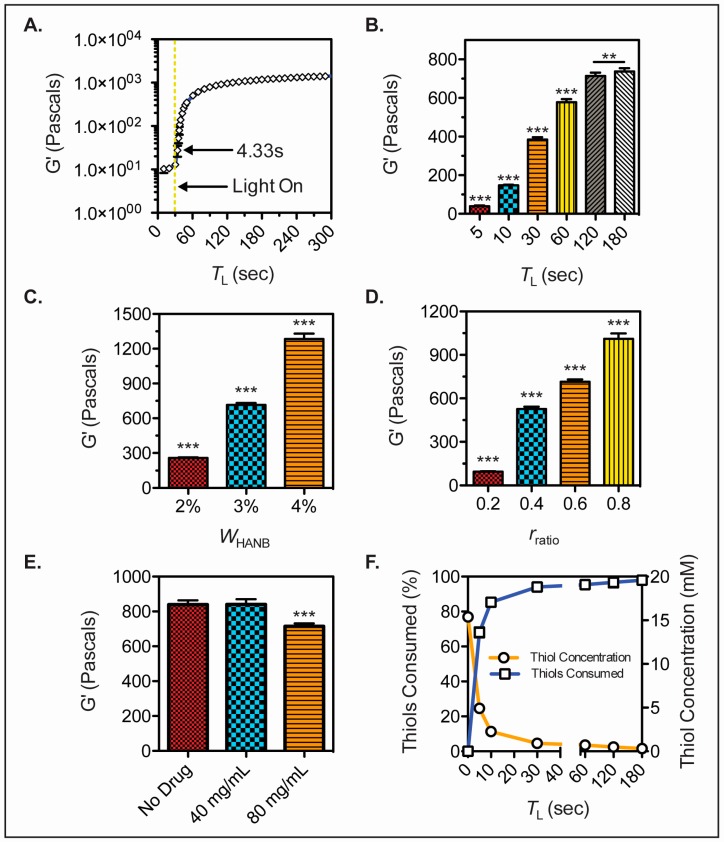
HANB hydrogel mechanical properties. (**A**) In-situ photorheology of hydrogel solution (*W*_HANB_ = 3%, *r*_ratio_ = 0.6, *T*_L_ = 2 min) indicating gelation kinetics; (**B**) G’ of hydrogels (*W*_HANB_ = 3%, *r*_ratio_ = 0.6) exposed to different visible light exposure times; (**C**) G’ of hydrogels (*T*_L_ = 2 min, *r*_ratio_ = 0.6) with varying *W*_HANB_; (**D**) G’ of hydrogels (*W*_HANB_ = 3%, *T*_L_ = 2 min) with varying *r*_ratio_; (**E**) G’ of hydrogels (*W*_HANB_ = 3%, *r*_ratio_ = 0.6, *T*_L_ = 2 min) with increasing Ropinirole HCL concentrations; (**F**) Results of Ellman’s reagent test on thiol concentration over time (*W*_HANB_ = 3%, *r*_ratio_ = 0.6, *T*_L_ = 2 min). All the bioink formulations contained 80 mg/mL of Ropinirole HCL, with the exception of (**E**). Asterisks denote statistical significance (** denotes *p* < 0.01; *** denotes *p* < 0.001).

In situ photorheology was carried out to assess the gelation kinetics of the bioink. Hydrogel precursor solution was placed in a rheometer and exposed to light after 30 s of G’ monitoring. The bioink experienced fast gelation, achieving a soft hydrogel in less than 5 s of light exposure (Figure 2A). Moreover, the G’ of hydrogels with *T*_L_ = 5, 10, 30, 60, 120, and 180 s was measured and a statistical difference was observed between gels cured for up to 3 min of light exposure time (Figure 2B). The effect of HANB weight percent and crosslinking ratio over the storage modulus of the gels was analyzed (Figure 2C,D). Results demonstrate that HANB weight percent has a stronger effect on storage modulus when compared to crosslinking ratio, with a *W*_HANB_ = 4% achieving G’ values over 12,000 Pascals. However, increasing HANB weight percent within the bioink represents a direct increase in the viscosity of the solution, whereas the addition of PEGDT for the increase of crosslinking ratio has a lower effect on viscosity, allowing for increases in hydrogel strength without detrimentally affecting the bioink’s droplet formation capacity. The effect of API concentration on G’ was assessed by making hydrogels with Ropinirole HCL at concentrations of 0, 40, and 80 mg/mL (Figure 2E). A statistical difference was found between the 80 mg/mL hydrogels and the less concentrated conditions, where higher API concentrations resulted in lower G’ values. These results indicate that the mechanical properties of the hydrogel remain stable at low dosages, indicating higher consistency over mechanical stability when loading potent drugs that achieve their therapeutic effect at low quantities.

The swelling ratio of the hydrogel was measured by taking the ratio of the hydrogel swollen state mass over the dry mass. A swelling ratio of 21.14 was obtained, denoting the water absorption ability of the hyaluronic acid matrix (Table 1).

An Ellman’s reagent test was performed on hydrogels with *T*_L_ = 5, 10, 30, 60, 120, and 180 s, in order to quantify the thiol conjugation within the bioink as the step growth reaction occurs. A drastic decrease in thiol concentration was observed within 30 s of light exposure with over 90% of the thiols being conjugated, validating the fast reaction kinetics of the bioink (Figure 2F).

A critical characteristic of the bioink design is the ability for droplet formation and inkjet printability. To quantify this, the inverse of the Ohnesorge number (*Z*) was determined, which relates the viscosity, surface tension, and density parameters of a substance [18]. Materials with *Z* values within the range 4 ≤ *Z* ≤ 14 are considered printable fluids, where values above 14 experience strong viscous forces that prevent droplet formation and values below 4 experience the formation of satellites. Table 1 shows the values for the density, surface tension, viscosity, and nozzle diameter of the piezoelectric nozzle used to dispense the bioink. These values resulted in a *Z* value of 7.82, which falls in the category of printable fluids.

**Figure 3:**
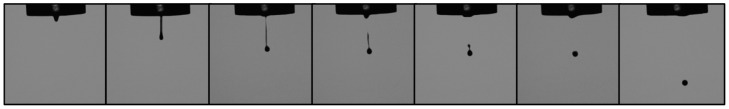
Bioink droplet formation picture sequence.

The droplet formation was further tested in an 80 µm piezoelectric nozzle, with driver settings at 45 V, a pulse width of 15 µs, and a frequency of 2000 Hz. Under these parameters, consistent droplet formation and dispensing was achieved (Figure 3), with a continuous printing time of at least 1 h and an idle time of 1 min between prints.

### 3.3. Preform Tablet Fabrication

**Figure 4:**
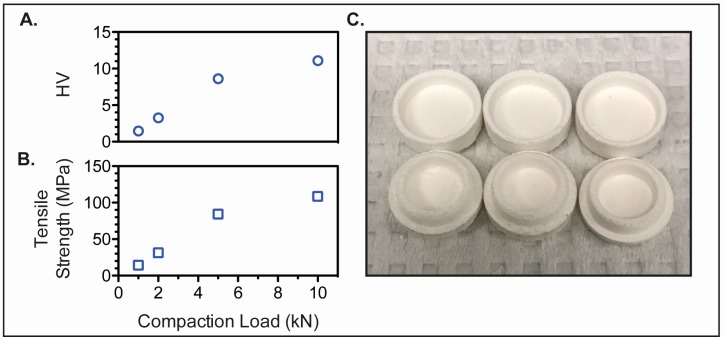
Mechanical properties of blank preform tablets. (**A**) Hardness (HV) and (**B**) tensile strength (MPa) of microcrystalline cellulose preform tablets; (**C**) Fabricated preform tablets.

The hardness (Figure 4A) and tensile strength (Figure 4B) of custom preform tablets made of microcrystalline cellulose using direct powder compression increased with compression force, and at least 5 kN compression force was required to produce tablets with adequate mechanical strength and handling properties (Figure 4C). To facilitate the rapid disintegration of the preform tablet, croscarmellose sodium was added to the preform tablet at 5% (*w*/*w*) [24]. Addition of the super disintegrant to the preform tablet did not greatly impact the mechanical properties of the tablet (10 kN compression force; HV = 9; Tensile Strength = 88 MPa). The advantages of using microcrystalline cellulose as the diluent include its good compressibility, as well as compactibility due to its plastic deformation and strong hydrogen bonding among hydroxyl groups [25]. This was particularly important for the successful formation of the positive features of the preform tablet. Furthermore, it is broadly compatible with APIs and physiologically inert [26]. However, microcrystalline cellulose also acts as a disintegrant and the printing of hydrophilic bioinks directly into the preform tablet causes swelling and the tablet to break apart. As such, a thin hydrophobic coating (Eudagrit(R) E100) was added to the preform tablet to maintain the integrity of the preform tablet while the bioink was being printed. A 20% (*w*/*w*) Eudagrit(R) E100 solution in acetone was required to prevent tablet swelling during the printing time. However, a reduction in printing time achieved by increasing the number of inkjet nozzles dispensing bioink loaded with API would reduce the contact time between the water-based bioink and the preform tablet. Accordingly, lower concentrations of polymer coating could be used to line the preform tablet well.

### 3.4. Tablet Fabrication and Dissolution Test

**Figure 5:**
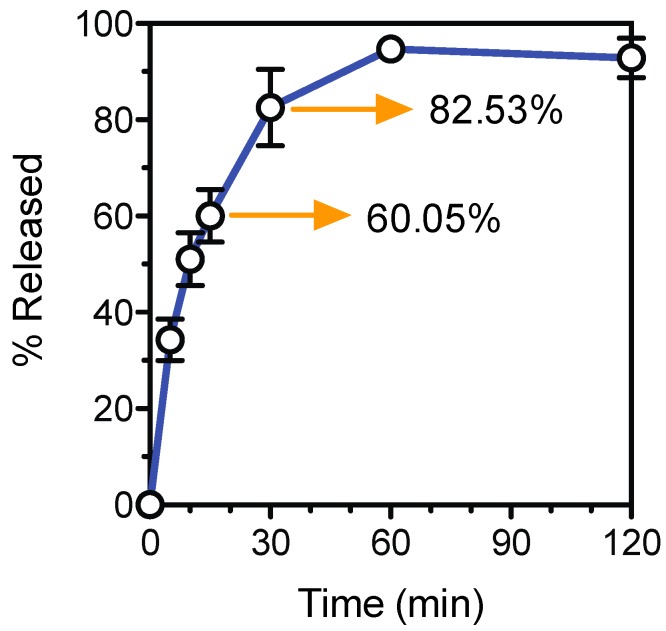
Ropinirole HCL release profile.

The tablet formation scheme designed in this study allows for the manufacture of dosage controlled tablets at fast manufacturing times. A Ropinirole HCL dose of 5.7 mg was dispensed into a blank preform tablet with an accuracy of 92.87% ± 4.08%. Drug release data (Figure 5) shows that 60% of the Ropinirole HCL was released within the first 15 min of dissolution and over 80% was released in 30 min. The preform tablet was designed to undergo rapid dissolution under acidic aqueous mediums. This preform tablet property and the high permeability of the hydrogel result in quick drug release kinetics applicable to API requiring such release profiles for fast therapeutic effects.

**Figure 6:**
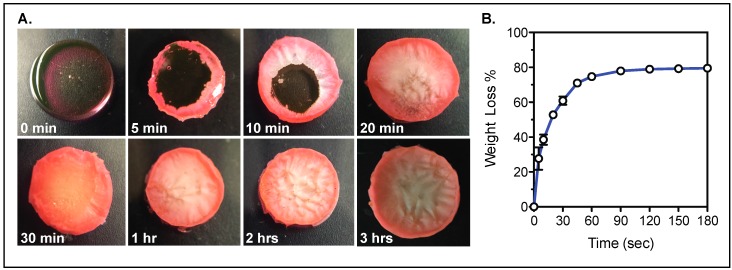
Hydrogel water content removal. (**A**) Hydrogels exposed to 50 °C for varying times; (**B**) Percent of weight loss in the hydrogel due to water removal with increasing drying times.

To extend drug stability and tablet shelf life, the tablet would require removal of the water content. Drying tests of the hydrogels show that the water can be removed from the hydrogel in 1.5 h at a temperature of 50 °C (Figure 6, Figure S3).

The photocurable bioink developed here could potentially be used for PolyJet 3D printing [27,28], where the liquid is dispensed under constant light exposure to create solid materials with diverse shapes and mechanical properties. Improvements on the mechanical strength of pharmaceutical hydrophilic photocurable bioinks would allow for their application in polyjet technology, and eliminate the need for blank preform tablets. Additionally, future work using multiple nozzles simultaneously would further improve the printing speed of pharmaceutical tablets over other 3D Pharming techniques, such as powder bed 3D printing and fused deposition modelling. The combination of fast printing speeds with rapid polymerization upon short exposures to light results in the rapid production of pharmaceutical tablets for immediate consumption. This would be exceptionally advantageous for APIs with a short shelf life. Finally, more research is required in the development of hydrophilic bioinks capable of incorporating large drug dosages.

## 4. Conclusions

This study reports the synthesis of a norbornene modified hyaluronic acid photocurable bioink for the inkjet dispensing of hydrophilic API and subsequent manufacture of pharmaceutical tablets. The bioink was engineered to undergo polymerization through a thiol-ene reaction upon exposure to visible light. The mechanical tunability of the bioink was demonstrated by manipulating the HANB weight percent, crosslinking ratio, and light exposure time. The drug loaded bioink was dispensed through an inkjet piezoelectric nozzle into blank preform tablets made by direct powder compression, specifically designed to undergo rapid dissolution when exposed to acidic aqueous mediums. Drug release studies revealed a 60% Ropinirole HCL release within the first 15 min of tablet dissolution. This work demonstrates the utility of inkjet printing for the 3D Pharming of hydrophilic APIs.

## Figures and Tables

**Table 1:** Swelling ratio and parameters for *Z* value analysis of bioink.

Nozzle Diameter (mm)	Density (kg/m^3^)	Surface Tension (mN/m)	Viscosity (mPa·s)	*Z*	Swelling Ratio
0.08	1042	51.47	8.38	7.82	21.14

## Supplementary Materials

The following are available online at http://www.mdpi.com/2306-5354/4/1/11/s1, Figure S1: NMR shifts of two-step HANB synthesis pathway, Figure S2: Custom preform tablet punches and die for direct powder compression, Figure S3: Statistics on tablet water removal percentage over drying time.
