# Supplementary material for: Photocurable Bioink for the Inkjet 3D Pharming of Hydrophilic Drugs
Source: Bioengineering (Basel). 2017 Jan 28;4(1):11. doi: 10.3390/bioengineering4010011 (PMC5590429; doi:10.3390/bioengineering4010011)
Supplement: Supplementary file 1 [file bioengineering-04-00011-s001.pdf]

Supplementary Materials: Photocurable Bioink for the Inkjet 3D Pharming of Hydrophilic Drugs

Giovanny F. Acosta-Vélez, Chase S. Linsley, Madison C. Craig and Benjamin M. Wu

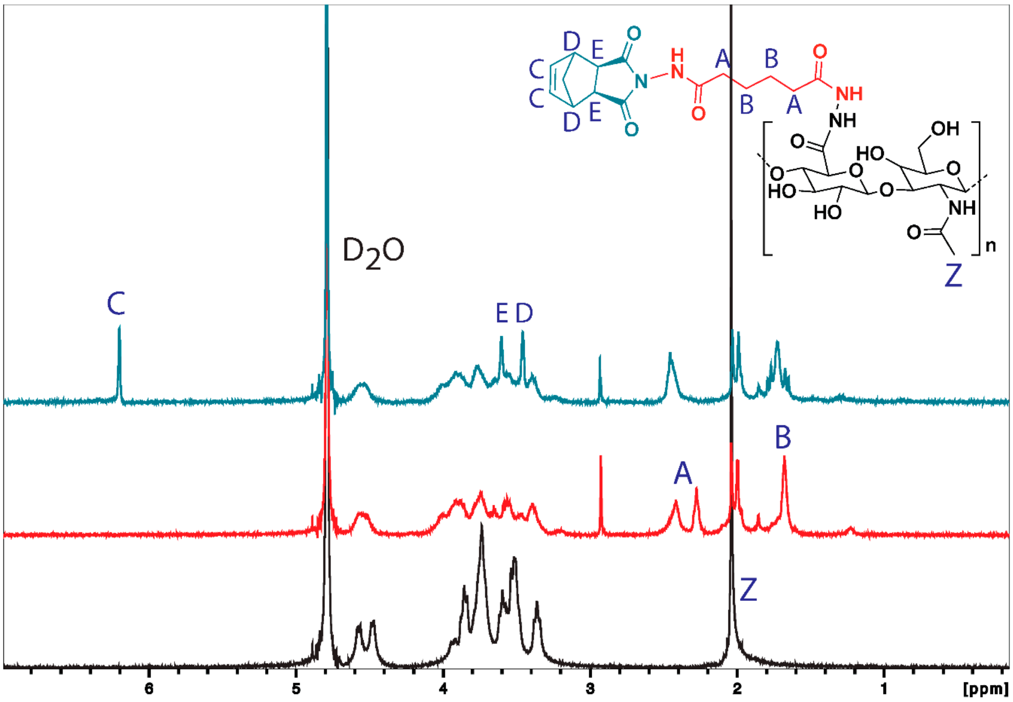

Figure S1. ^{1}H NMR shifts of norbornene groups (D_2O): **Z** (methyl protons in hyaluronic acid, 3H); **A** (α protons in the ADH group, 4H); **B** (β protons in the ADH group, 4H); **C** (*endo* vinyl protons, 2H); **D** (bridgehead protons, 2H); **E** (norbornene α protons, 2H).

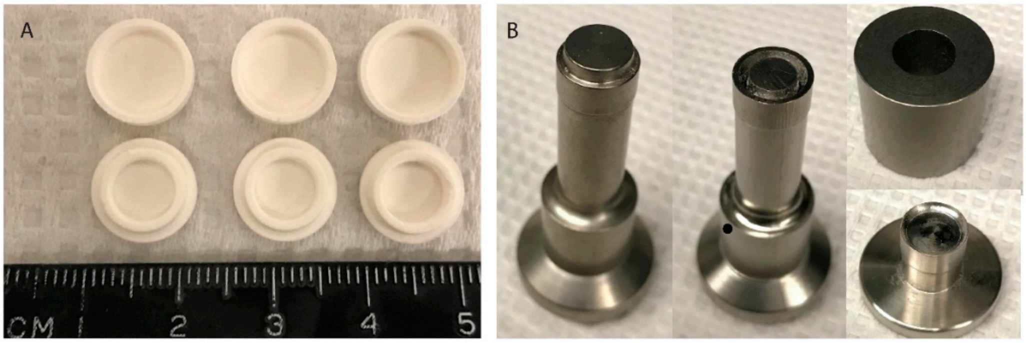

Figure S2. Custom preform tablet punches and die for direct powder compression.

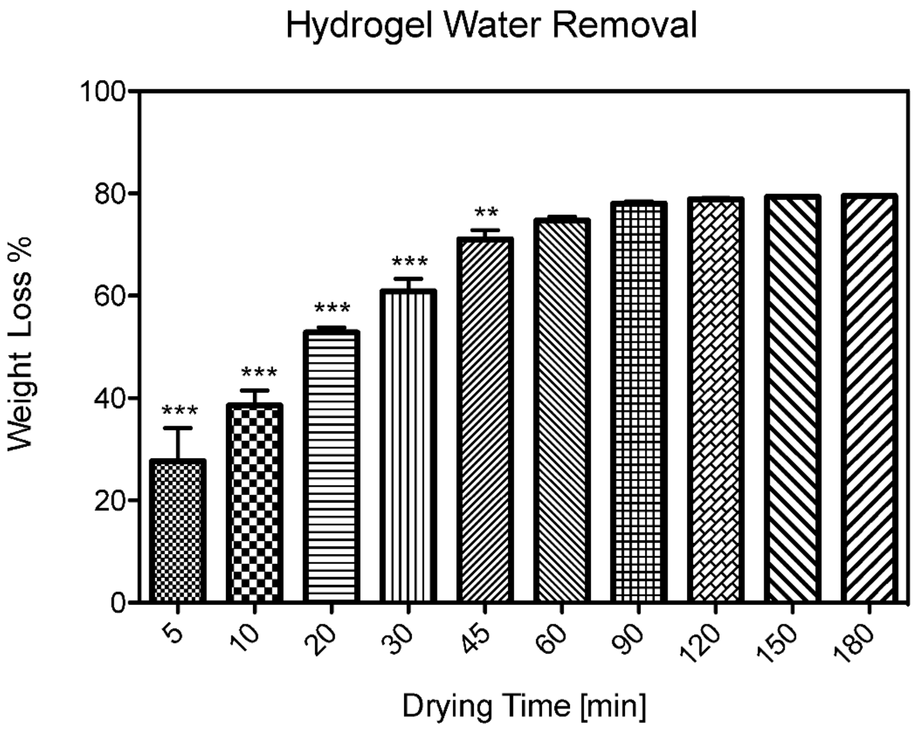

Figure S3. Statistics on tablet water removal percentage over drying time. A One-way ANOVA test with a Dunnett's post-test analysis was done, with column (180 min) taken as control. Asterisks denote statistical significance (** denotes $p < 0.01$; *** denotes $p < 0.001$.). Results show no statistical difference after 60 min of drying at 50 °C.
